# Sperm freezing damage: the role of regulated cell death

**DOI:** 10.1038/s41420-024-02013-3

**Published:** 2024-05-18

**Authors:** Erhan Hai, Boyuan Li, Jian Zhang, Jiaxin Zhang

**Affiliations:** Inner Mongolia Key Laboratory of Sheep & Goat Genetics Breeding and Reproduction, College of Animal Science, Inner Mongolia Agricultural University, Hohhot, 010018 Inner Mongolia China

**Keywords:** Cell death, Reproductive biology

## Abstract

Substantial progress in research on sperm cryopreservation has occurred since the twentieth century, especially focusing on improving sperm freezing procedures and optimizing semen extenders. However, the cellular biological mechanisms of sperm freezing damage are still unclear, which greatly restricts the promotion and development of sperm cryopreservation. An essential component of sperm freezing damage is the occurrence of cell death. Considering the existence of multiple types of cell death pathways, this review discusses connections between characteristics of regulated cell death (e.g., apoptosis and ferroptosis), and accidental cell death (e.g., intracellular ice crystals) with sperm freezing damage and explores possible future research directions in this field.

## Facts

- The main type of cell death in sperm freezing damage remains to be determined.
- Oxidative stress is one of the most important causes of sperm freezing damage.
- Oxidative stress can induce various types of regulated cell death (RCD), including apoptosis and ferroptosis.

## Open questions

- Which types of RCD are driven by oxidative stress during sperm cryopreservation?
- If multiple types of RCD appear during sperm cryopreservation, which one is most important?
- What are the biological pathways of critical RCD?

## Introduction

**Fig. 1:**
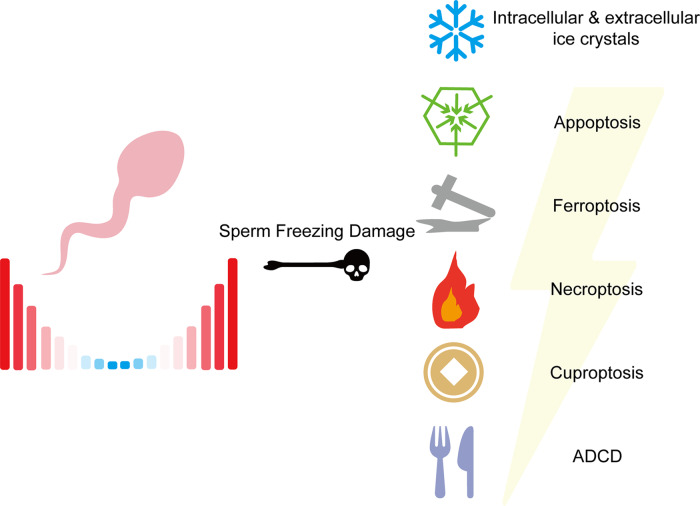
Types of cell death that may occur during sperm cryopreservation. The occurrence of ACD due to intracellular ice crystal formation has potentially been avoided with the widespread application of glycerol. Among RCD, apoptosis is the only type that has been definitively identified in sperm cryopreservation damage, although its inductive mechanisms remain elusive. Although the term “ferroptosis” has not yet been explored in sperm cryopreservation research, it may be highly relevant to sperm freezing damage. Additionally, as oxidative stress is also associated with necroptosis, cuproptosis, and ADCD, these represent potential areas of investigation in sperm freezing damage research.

Sperm are more prone to death than other types of cells, especially during cryopreservation [1]. This is due to their highly dense chromatin, which cannot respond to changes in the microenvironment and other factors to generate genomic responses that maintain important cellular functions, such as ATP synthesis and the maintenance of redox homeostasis, and protect the integrity of the plasma membrane [2]. Cell death can be classified as accidental cell death (ACD) or regulated cell death (RCD). ACD refers to the virtually instantaneous and uncontrollable form of cell death corresponding to the physical disassembly of the plasma membrane caused by extreme physical, chemical, or mechanical cues. RCD refers to cell death that results from the activation of one or more signal transduction modules and, hence, can be pharmacologically or genetically modulated (at least kinetically to some extent) [3]. During the cryopreservation process, sperm may experience cell death caused by intracellular ice crystals, oxidative stress, and other factors, collectively referred to as sperm freezing damage [4–7]. Therefore, according to the classification of cell death types, sperm freezing damage can be divided into ACD caused by intracellular ice crystals and RCD induced by oxidative stress [1] (Fig. 1).

## Relationship between accidental cell death and sperm freezing damage

Physical damage to the sperm plasma membrane caused by intracellular ice crystals is considered one of the main factors that contribute to the poor quality of frozen-thawed semen [1, 8–12]. The control of dehydration and application of osmoprotectants in sperm cryopreservation are the main measures used to delay this event (Fig. 1).

### Control of dehydration

Conventional cryopreservation is the most widely used sperm cryopreservation technique and includes manual and programmable freezing. In the manual freezing technique, 0.25- or 0.5-mL plastic straws filled with extended semen are placed in contact with liquid nitrogen (LN_2_) vapor 4–5 cm above the LN_2_ for no longer than 15 min (10 min is recommended) before being immersed in LN_2_ for storage [11]. Programmable freezing is a method of gradually freezing/cooling sperm in two or three steps within 2 to 4 h using a programmable biofreezer [13].

The formation of intracellular ice crystals depends on the cooling rate. During the slow cooling process, sperm dehydration is controlled by the increase in osmotic pressure caused by extracellular ice crystals. Excessive dehydration can cause unexpected sperm death (osmotic damage, structural loss), called the “solution effect” [14]. In addition, extracellular ice crystals may cause physical damage to sperm [8]. A faster cooling rate can prevent sperm from dehydrating, which can lead to the formation of intracellular ice crystals, which is fatal to sperm [15]. Therefore, a suitable cooling rate is particularly important for sperm and requires a balance between the sperm dehydration rate and the formation of intracellular ice crystals during sperm freezing, which is known as the “two-factor hypothesis” [10].

### Permeable cryoprotectants

For many cell types, including mammalian oocytes and embryos, the osmotic behavior of cells during freezing can be predicted from numerical models. Therefore, the probability of intracellular ice crystal formation under different linear cooling rates can be estimated [15]. This model suggests that cell freezing damage is caused by the formation of intracellular ice crystals. By combining cryomicroscopy with observation, it is possible to visualize the formation of intracellular ice crystals in oocytes and embryos subjected to different cooling rates [14]. However, experimental observations of mammalian sperm cryopreservation with glycerol are inconsistent with model prediction results [16–20].

The application of glycerol as a permeable cryoprotectant marks a significant advance in semen cryopreservation [21]. Similar to other permeable cryoprotectants, glycerol can undergo hydration reactions with water-based solvents, increasing the viscosity of intracellular fluids and thereby inhibiting the formation of ice crystals to a certain extent and protecting cells [22]. Morris et al. [23, 24] observed the ultrastructure of frozen human and horse semen subjected to cooling rates ranging from 0.3 to 3000 °C/min using freeze-fracture electron microscopy and freeze substitution. Intracellular ice crystals were not detected at any cooling rate, and glycerol played a major role in this process.

## Regulated cell death and sperm freezing damage

However, 40–50% of sperm still die after freezing, and surviving sperm subpopulations after thawing are also affected and are unable to perform normal physiological functions [13, 25–28].

The development of sperm cryopreservation technology has significantly improved the quality of frozen semen and prevented a large proportion of sperm ACD caused by intracellular ice crystal formation [10, 21–24]. However, approximately 40–50% of sperm still experience mortality following cryopreservation. Furthermore, the surviving subpopulations of sperm demonstrate functional impairments upon thawing, compromising their ability to perform normal physiological functions [13, 25–28]. Interestingly, fresh semen (with a total motility of over 70–80%) that is considered of sufficient quality for freezing also has a mortality rate of approximately 20% after ejaculation and has significant individual [29, 30] and seasonal differences in freezability [31, 32]. In addition, the freezability of semen is not only related to the sperm itself but also to the seminal plasma [33]. These findings indicate that sperm are highly vulnerable to death, and a certain development process exists that may be accelerated by freezing. Importantly, this process can be modulated, and its characteristics do not meet the definition of ACD. Therefore, we speculate that RCD plays an important role in sperm freezing damage.

### RCD regulated cell death

The first scientific observation of RCD occurred in 1842 when Karl Vogt noticed that the notochord of tadpoles disappeared during development and found that the disappearance of such cells had physiological significance during the developmental stage. When the term “apoptosis” emerged in 1972, research on RCD began to increase [34]. In the twenty-first century, multiple types of non-apoptotic RCD were discovered, such as necroptosis [35] and ferroptosis [36].

There are two distinct forms of RCD, although the underlying molecular mechanisms exhibit considerable overlap. RCD is involved in two diametrically opposed scenarios. On one hand, RCD can occur in the absence of any exogenous environmental perturbation, hence operating as a built-in effector of physiological programs for development or tissue turnover. These completely physiological forms of RCD are generally referred to as programmed cell death [3, 37, 38]. On the other hand, RCD can originate from perturbations of the intracellular or extracellular microenvironment that are too intense or prolonged for adaptative responses to cope with the stress and restore cellular homeostasis [39]. In general, cells will trigger one or more types of RCD in response to different stressors, especially oxidative stress [40].

### Sperm freezing damage and oxidative stress

Oxidative stress is one of the most important causes of sperm freezing damage, resulting in damage to the structural and functional integrity of sperm. Its essence is an imbalance of intracellular oxidation-reduction reactions [41, 42]. Reactive oxygen species (ROS) are a class of highly reactive oxidative free radicals that are produced by normal physiological processes and play an important role in cell signaling and tissue homeostasis [43]. The production of ROS in mature sperm primarily occurs through two distinct pathways. One pathway involves the nicotinamide adenine dinucleotide phosphate (NADPH) oxidase system, which is located on the sperm plasma membrane. The other pathway is associated with electron leakage from the mitochondrial electron transport chain, which serves as the primary source of ROS production in sperm [8, 44–46]. The role played by ROS depends on their concentration in sperm; only when the concentrations are at an appropriate level can sperm exert normal physiological functions [47]. Under physiological conditions, ROS ensure the stability of chromatin and protect DNA from damage during sperm development and maturation. Additionally, ROS can activate the cAMP pathway and its downstream signaling cascade, which is important for sperm capacitation and forward movement [41, 43, 45, 46]. However, when the ROS level increases, sperm undergo oxidative stress, causing plasma membrane lipid peroxidation and mitochondrial damage. Lipid aldehydes produced by lipid peroxidation bind to proteins in the mitochondrial electron transport chain, triggering an increase in ROS in a self-perpetuating cycle and further causing DNA damage, which is referred to as “oxidative damage” [41, 43, 47–49].

The oxidative stress experienced by sperm during freezing can be divided into two aspects. The first aspect is the consumption of antioxidants. Sperm support their movement through a high mitochondrial metabolic rate. Due to the extreme differentiation of sperm cells, the highly dense chromatin cannot produce an antioxidant response in the genome. Additionally, during the initial support of cells, most sperm cell cytoplasm is absorbed, resulting in a lack of cytoplasmic antioxidants, including enzymes and small-molecule ROS scavengers [2]. The second aspect pertains to the disruption of the extracellular microenvironment. To accommodate changes within this microenvironment during the freezing process, including cooling and dehydration, sperm decrease their metabolic rate, thereby enhancing their chances of survival. However, during the freezing and thawing stage, as the microenvironment conditions and metabolic rate recover, ROS surge during a short period of time, and if not controlled, sperm will undergo oxidative stress and eventually die [8, 46, 50]. Oxidative damage can induce various types of RCD [3, 40, 51, 52], including apoptosis [53, 54] and ferroptosis [36, 55, 56]. Oxidative damage is not only the cause of various types of RCD, but also results from RCD.

### Sperm freezing damage and apoptosis

Apoptosis is the only RCD marker of sperm freezing damage [57]. Many studies show that changes in apoptotic markers, such as activation of the caspase family, phosphatidylserine (PS) externalization, and the mitochondrial membrane potential, decrease during sperm cryopreservation [4, 25, 58–64] and may involve both intrinsic and extrinsic apoptotic pathways [1, 9, 57, 60, 65, 66]. These two pathways can operate independently, yet also demonstrate interconnectedness.

#### Apoptosis

##### Extrinsic apoptosis

There are two main receptors for apoptosis on the cell membrane, FAS and tumor necrosis factor (TNF)-related apoptosis-inducing ligand receptor (TRAIL-R), which are associated with the FAS-associated via death domain (FADD) [67–70]. When FAS and TRAIL-R bind to their ligands (FAS-L and TRAIL), conformational changes occur, which further lead to conformational changes in FADD. The altered conformation of FADD causes the precursor state of Caspase 8/10 to mature, and the activated Caspase 8/10 further activates Caspase 3/7 to induce apoptosis and cleave the BH3 interacting domain (Bid) death agonist to form a truncated Bid (t-Bid) [71–74].

##### Intrinsic apoptosis

t-Bid binds to the mitochondrial membrane, activating the BCL2-associated X (BAX) apoptosis regulator and BCL2 antagonist/killer 1 (BAK1; commonly known as BAK) located on the mitochondrial membrane [71, 72]. Bax and Bak are inhibited by pro-apoptotic and anti-apoptotic members of the BCL2 family and BCL2-like 1 (BCLXl). When Bax and Bak cannot be inhibited and are activated, they induce mitochondrial outer membrane permeabilization (MOMP) and release cytochrome C [75–77]. Cytochrome C binds to the precursor of Caspase 9 and apoptotic peptidase activating factor 1 to form an apoptotic body. Such apoptosomes can induce the maturation of Caspase 9 and further activate Caspase 3/7, leading to cell apoptosis [78].

#### Potential factors inducing apoptosis in sperm freezing damage

**Fig. 2:**
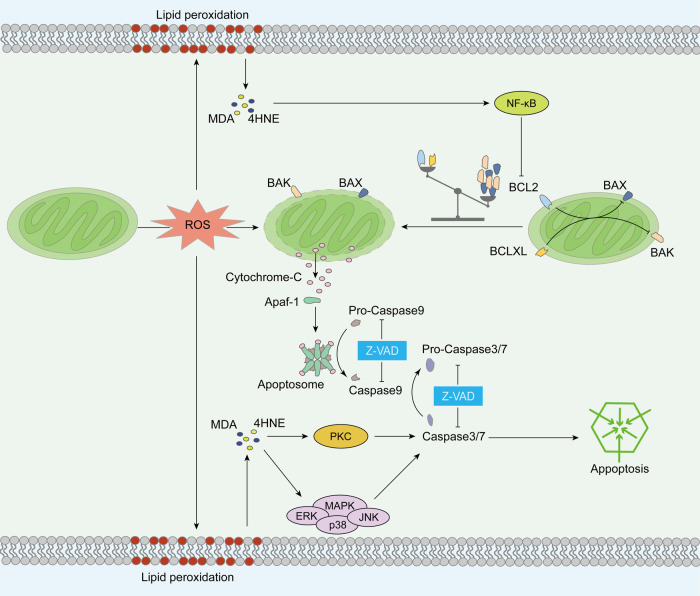
Potential mechanisms of apoptosis in sperm freezing damage. Currently, the triggering factors of apoptosis during sperm cryopreservation remain elusive. We hypothesize that they may be associated with oxidative stress in mitochondria and the products of lipid peroxidation.

Although apoptosis has been confirmed to occur in sperm freezing damage, the specific regulatory mechanisms remain unclear. Notably, the activation of Caspase 3 during sperm cryopreservation is highly correlated with the degree of lipid peroxidation [79, 80]. Therefore, in this section, we discuss the potential mechanisms underlying lipid peroxidation-induced apoptosis during this stage (Fig. 2). ROS elevation induces cardiolipin peroxidation on the inner mitochondrial membrane (IMM), leading to the separation of cytochrome C and its release. The IMM also regulates MOMP through the mitochondrial permeability transition pore (mPTP), releasing cytochrome C and activating downstream cascades to complete apoptosis [81]. In addition, lipid peroxidation products regulate apoptosis by activating different signaling pathways, including the nuclear factor kappa B (NF-κB), mitogen-activated protein kinase (MAPK), and protein kinase C (PKC) signaling pathways. The NF-κB family is widely involved in inflammation, cell death, and the stress response [82]. Lipid peroxidation products can inhibit the degradation of I kappa B to maintain NF-κB activity, and NF-κB can phosphorylate the anti-apoptotic protein Bcl-2, rendering it inactive during lipid peroxidation [83, 84]. The MAPK signaling pathway is responsible for cell signaling in response to various stimuli, including oxidative stress [85]. Lipid peroxidation products can form adducts with extracellular regulated kinases, c-Jun-N-terminal kinases, and p38, which activate MAPKs, induce caspase maturation, and initiate the apoptosis process [86, 87]. The PKC signaling pathway is a key factor regulating cell signaling transduction, such as cell proliferation, differentiation, and apoptosis [88]. Lipid peroxidation products can activate PKCδ, which is further cleaved by Caspase 3 to generate a constitutively active catalytic fragment, amplifying the apoptotic cascade reaction [89].

#### Apoptosis is not the only evidence of RCD in sperm freezing damage

Oxidative stress is one of the most important causes of sperm freezing damage. However, in previous studies, researchers only focused on the impact of apoptosis on sperm during this period, ignoring other types of RCD. This limitation may also be one of the reasons for the slow development of sperm cryopreservation technology. Apoptosis is the only RCD marker of sperm freezing damage. However, some studies suggest that apoptosis may not be the main factor that causes death in frozen-thawed sperm.

Z-VAD-FMK (Z-VAD) is a pan-caspase irreversible inhibitor that inhibits RCD caused by the caspase family, including apoptosis [90]. Ideally, Z-VAD could markedly reduce the death indicators of frozen-thawed semen, including improved vitality and plasma membrane integrity. However, the addition of Z-VAD before freezing had no significant effect on the integrity of the plasma membrane of frozen-thawed bovine sperm, and the addition of Z-VAD after thawing had no significant effect on the viability of bovine sperm [91]. In addition, similar conclusions were drawn from the frozen semen of dogs; regardless of whether Z-VAD was added before or after freezing, sperm motility and plasma membrane integrity were not improved [92]. Annexin V/propidium iodide (PI) double staining is a commonly used method for detecting apoptosis [93] and is widely used in studies of sperm freezing damage [94]. Annexin V can bind to PS, and PI is a nucleic acid dye that only enters cells when the cell membrane is damaged. Therefore, the Annexin V/PI double staining method can label early apoptotic cells (Annexin V+, PI−) and late apoptotic cells (Annexin V+, PI+). However, when cells undergo a type of regulated necrosis other than apoptosis, only early apoptotic cells can be used as apoptotic markers. This is because when regulated necrosis occurs with loss of plasma membrane integrity, Annexin V enters the cell and binds to PS [95, 96]. Therefore, the sperm sorted by Annexin V immunomagnetic beads (Annexin V+) [97, 98] may not be entirely apoptotic. In addition, the decrease in mitochondrial membrane potential in frozen-thawed sperm may not be entirely caused by apoptosis, and necrostatin-1 can also rescue the decrease in mitochondrial membrane potential induced by oxidative stress [99]. Moreover, DNA fragmentation, as a marker of late apoptosis, does not show significant changes during sperm cryopreservation and thawing [60, 100]. It is important to clarify that, whereas lipid peroxidation may trigger sperm apoptosis, the process of apoptosis itself does not induce lipid peroxidation [55].

### Sperm freezing damage and ferroptosis

The response to oxidative stress is a key pathway determining the fate of cells. Among the factors that cause oxidative stress in cells, oxidative modification of lipids in biological membranes, especially lipid peroxidation, is an important regulator of cell fate. Widespread lipid peroxidation leads to cell death through a type of RCD called “ferroptosis” [101]. Although the term “ferroptosis” has not yet been mentioned in studies of sperm freezing damage, sperm possess many prerequisites for inducing ferroptosis, and changes in some key indicators are also reported in relevant research.

#### Ferroptosis

Ferroptosis is a newly discovered type of RCD characterized by a lethal level of iron-dependent lipid peroxidation, which is associated with the oxidation of polyunsaturated fatty acids bound to phospholipids (PUFA-PLs) on the biological membrane [36, 55, 56]. Oxidation-reduction and iron regulation comprise the central framework of ferroptosis [55], which is independent of the caspase family and the necrosome, and manifest as a necrotic morphology [36]. The resistance of cells to ferroptosis is mainly reflected by their ability to scavenge membrane lipid peroxides. Solute carrier family 7member 11 (SLC7A11)-reduced glutathione (GSH)-glutathione peroxidase 4 (GPX4) is the main regulator of ferroptosis [55].

GPX4 is a selenoprotein that uses GSH to reduce oxidized PUFA-PLs and inhibit ferroptosis [102]; the synthesis of GSH requires the uptake of cystine by SLC7A11. Therefore, GPX4 and SLC7A11 are also major targets for inducing cell ferroptosis [36, 103, 104]. In addition, independent anti-ferroptosis mechanisms of GPX4 have been discovered in recent years. Coenzyme Q_10_ (CoQ_10_) is the second endogenous mechanism of resistance to ferroptosis and exists throughout the entire biological membrane. Reduced CoQ_10_ can reduce lipid peroxides through self-oxidation, and then ferroptosis suppressor protein 1 regenerates reduced CoQ_10_ through NADPH [105, 106]. The oxidation of PUFA-PLs is driven by the formation of hydroxyl radicals through the Fenton reaction between Fe^2+^ and H_2_O_2_ [107, 108], which depends on the concentration of the labile iron pool (LIP) in cells [109]. Iron regulation is the key to ferroptosis. On one hand, Fe^3+^ binds to transferrin and enters the cell membrane via transferrin receptor 1, subsequently being released into the cytoplasm [110–116]. On the other hand, ferritin bound to Fe^3+^ forms autophagosomes via microtubule-associated protein 1 light chain 3 (LC3) and autophagy-related proteins 5 and 7 (ATG5/7) and combines with lysosomes to form autophagosomes. Relevant enzymes degrade proteins and release Fe^3+^, leading to an increase in LIP concentration and LIP accumulation, which triggers ferroptosis [117].

#### Key evidence of ferroptosis in sperm freezing damage

**Fig. 3:**
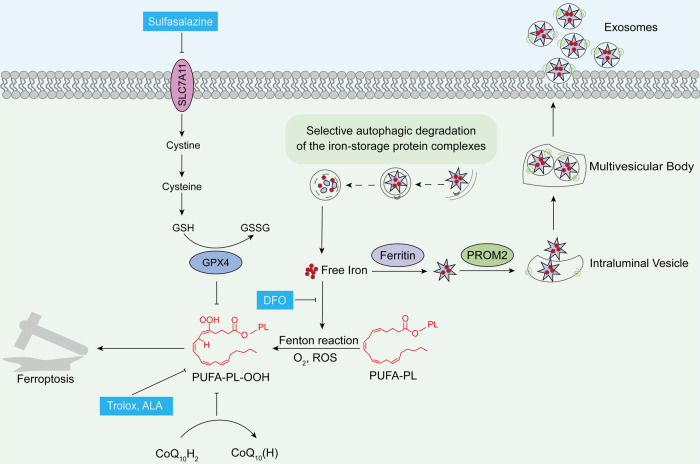
Potential mechanisms of ferroptosis in sperm freezing damage. The crucial regulatory components of ferroptosis, as well as the effects of ferroptosis-related inhibitors and activators on sperm, have been elucidated by numerous studies. Therefore, the role of ferroptosis in sperm freezing damage deserves further attention.

The mature sperm plasma membrane is abundant in polyunsaturated fatty acids (PUFAs), which not only maintain membrane fluidity but are also susceptible to oxidation. Consequently, membrane lipid peroxidation serves as a significant indicator of sperm freezing damage and may be associated with the Fenton reaction [118–122] (Fig. 3). Trolox and deferoxamine (DFO) are widely used inhibitors in ferroptosis research. Trolox can reduce lipid peroxides, whereas DFO chelates free iron ions, thereby decreasing LIP levels and suppressing the Fenton reaction [123]. The addition of Trolox to diluents safeguards the quality of frozen semen from both healthy individuals and patients with oligospermia [124] and also improves the plasma membrane integrity, acrosome integrity, and mitochondrial membrane potential of thawed rabbit semen [125]. Trolox also provides greater structural integrity (plasma membrane and mitochondria) and motility to frozen-stored ram spermatozoa [126]. In studies involving the induction of sperm oxidation models, DFO has been able to rescue sperm motility and reduce lipid peroxidation levels [127]. When Trolox and DFO are administered simultaneously, sperm motility parameters that are reduced during oxidative stress are significantly improved by up to 20% [128]. Additionally, α-lipoic acid (ALA) is an antioxidant widely used in sperm diluents that enhances the quality of thawed semen in humans [129, 130] and goats [131]. Interestingly, ALA is also considered a ferroptosis inhibitor, inhibiting ferroptosis by scavenging free radicals and chelating free iron ions [132, 133].

The redox and iron regulatory pathways of ferroptosis play crucial roles in the process of sperm cryopreservation (Fig. 3). The reduction of membrane lipid peroxides by GPX4 utilizing GSH is essential for thawed sperm survival [134]. The expression level of GPX4 in fresh semen can predict the quality of frozen sperm [135], and GSH content decreases during sperm freezing [136]. The synthesis of GSH in spermatozoa relies on the transport of cystine by SLC7A11 rather than the transsulfuration pathway [137]. Therefore, in addition to adding GSH to the diluent, the supplementation of cystine and cysteine can also significantly improve the quality of cryopreserved semen [137–141]. The beneficial effects of CoQ_10_ on sperm cryopreservation have been widely validated, particularly by a recent study demonstrating that thawed human spermatozoa exhibit a significant increase in necrotic cells that were rescued by exogenous CoQ_10_ addition [142]. Sun et al. [143] conducted a proteomic analysis of spermatozoa from dairy goats with different freezability and found that differentially expressed proteins were enriched in the ferroptosis pathway. Interestingly, ferritin expression was lower in the high-freezability group than in the low-freezability group. It is possible that when spermatozoa are exposed to oxidative stress, Prominin2 promotes the loading of ferritin into multivesicular bodies and its subsequent extracellular release via exosomes, serving as a mechanism driving cellular resistance to ferroptosis and avoiding severe ferroptosis levels [144].

Interestingly, research shows that dead sperm can be harmful to live sperm [1, 145, 146]. Therefore, sperm freezing damage may include the transmission of death from dead sperm in addition to environmental factors [146]. Obviously, this transmissible death is a type of RCD, but transmissibility is not a general characteristic of apoptosis [147–152], as it can only occur under certain circumstances [152–154]. In a model of cysteine starvation and SLC7A11 inhibition-induced ferroptosis, somatic and cancer cells exhibited comparable levels of ferroptosis propagation capacity [147], which adds weight to the importance of ferroptosis in sperm freezing damage.

### Sperm freezing damage and other types of RCD

**Fig. 4:**
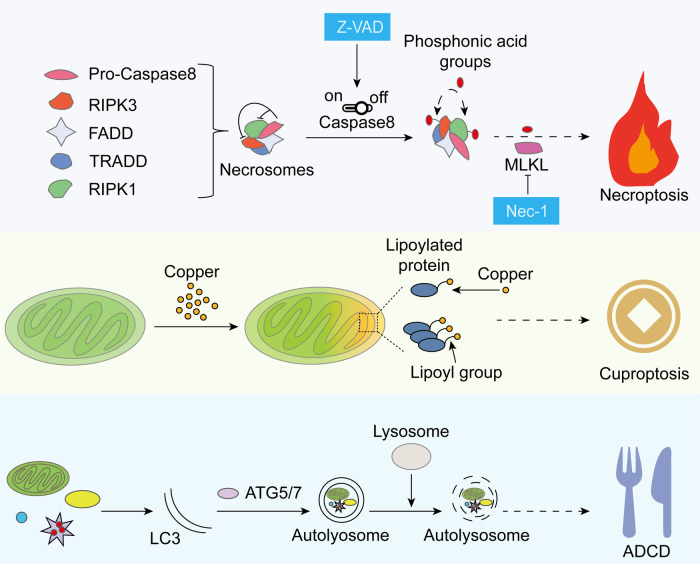
Potential mechanisms of necroptosis, cuproptosis, and ADCD in sperm freezing damage. Although the roles of necroptosis, cuproptosis, and ADCD in sperm freezing damage remain unclear, some studies suggest they could serve as potential targets for future research.

Current research on sperm freezing damage is still limited, and the underlying mechanisms remain to be elucidated. Given that oxidative stress is associated with various types of RCD in addition to apoptosis and ferroptosis, necroptosis, cuproptosis, and autophagy-dependent cell death (ACCD) may also be potential areas of investigation (Fig. 4).

#### Sperm freezing damage and necroptosis

Necroptosis is a type of regulated necrosis characterized by obvious necrosis morphology, including cell swelling, plasma membrane rupture, and spillover of intracellular components [3]. Activation of necroptosis is associated with receptor-interacting serine/threonine kinase 1 and 3 (R1PK1/3) and mixed lineage kinase domain-like pseudokinase (MLKL) [155–159]. Similar to apoptosis, necroptosis can also induce cell death by detecting disturbances in the intracellular and extracellular microenvironment through FAS and TRAIL-R [160, 161]. In in vitro experiments, low concentrations of H_2_O_2_ usually induce apoptosis. However, as the concentration of H_2_O_2_ increases, RCD shifts from apoptosis to necroptosis [162]. Considering the surge of ROS during sperm cryopreservation, we speculate that necroptosis is correlated with sperm freezing damage, but there is currently no relevant research to verify this speculation.

Caspase 8 serves as a switch between apoptosis and necroptosis. When Caspase 8 is activated, it inhibits the phosphorylation of RIPK1 and RIPK3, leading to apoptosis. However, when Caspase 8 is inactivated, RIPK1 and RIPK3 mutually activate their phosphorylation and subsequently activate downstream MLKL, triggering necroptosis [155, 156, 158, 163, 164]. Therefore, the inability of Z-VAD to rescue increased numbers of frozen-injured sperm may not be a dose-dependent problem [91] but may be due to the amplification of necroptotic signaling while inhibiting apoptosis [165, 166]. Moreover, sperms, as a type of highly metabolizing cell, produce a large amount of ROS in mitochondria, which is necessary for necroptosis [167]. Although there is currently no research on the effect of necroptosis on sperm freezing damage, necroptosis occurs in sperm in some male reproductive diseases. For example, varicocele, which causes male infertility, seriously affects semen quality, and the expression of RIPK1 and RIPK3 in the sperm of varicocele patients was significantly increased compared to a control group [168]. In addition, necrostatin-1 significantly improved the cryopreservation quality of spermatogonial stem cells [169], indicating that male germ cells are prone to necrotic apoptosis during cryopreservation.

#### Sperm freezing damage and cuproptosis

Similar to iron, copper ions play a crucial role as an integral component of cells and tissues in the male reproductive system. In relatively small quantities, copper serves as an essential cofactor for numerous biologically active molecules. However, its excessive accumulation can lead to metabolic disturbances, potentially compromising male fertility [170]. Cuproptosis, a type of cell death defined in 2022, primarily relies on the intracellular accumulation of copper ions. These ions directly bind to lipidated proteins involved in the tricarboxylic acid (TCA) cycle, resulting in their aggregation and dysfunction, which in turn disrupts the TCA cycle and triggers protein toxic stress, ultimately leading to cell death [171].

Certain studies on human sperm demonstrate a negative correlation between copper content in seminal plasma and sperm motility parameters [172, 173]. Knazicka et al. [174] point out that high doses of Cu^2+^ have a negative impact on the motility and mitochondrial activity of bull sperm. Roychoudhury et al. [175] also show that excessive copper sulfate inhibits the motility and membrane integrity of rabbit sperm, altering sperm morphology. Similarly, Rebrelo et al. [176] observed similar results in human sperm exposed to Cu^2+^ concentrations of 100 μg/mL. However, the occurrence of cuproptosis during sperm cryopreservation remains elusive. The degree of lipid peroxidation in sperm exhibits a dose-dependent relationship with copper ion levels [177], which is evidently associated with ferroptosis, suggesting two potential pathways. On one hand, copper ions can generate hydroxyl radicals through the Fenton reaction, driving lipid peroxidation [178]. On the other hand, copper ions can induce autophagic degradation of GPX4 to trigger ferroptosis [179]. Crucially, whether copper ion overload occurs in sperm freezing damage remains to be determined.

#### Sperm freezing damage and autophagy-dependent cell death

Autophagy is a catabolic process that degrades cytoplasmic substances through lysosomes, often serving as a cellular response mechanism to stress, particularly oxidative stress [180]. Autophagy is regarded as a double-edged sword, capable of protecting cell survival by eliminating damaged organelles yet also potentially leading to cell death [181]. The role of autophagy in cell death can be categorized into two types: 1) ACCD, which relies on autophagic mechanisms and manifests as unrestricted degradation of cellular contents leading to cell disruption, belonging to RCD; and 2) autophagy-mediated cell death (AMCD), which depends on other types of RCD and serves as a foundation for initiating other types of RCD [3, 182].

LC3 is a crucial component of the autophagy pathway. Upon autophagy activation, LC3-I is lipidated and converted into LC3-II. The ratio of LC3-II/LC3-I is widely used as a marker of autophagy activation [181]. Under environmental stress conditions such as incubation with H_2_O_2_, cooling at 4 °C, and the freeze-thaw process, the ratio of LC3-II/LC3-I in sperm is upregulated, indicating the activation of autophagy [183]. Interestingly, autophagy plays different roles under different oxidative stress conditions. Blocking autophagy in sperm exposed to H_2_O_2_ leads to deterioration in sperm quality and metabolic parameters, as well as an increase in cell death markers [184]. On the other hand, the use of autophagy inhibitors such as chloroquine and 3-AM significantly improves the survival rate of sperm stored at 4 °C for 96 hours and cryopreserved in liquid nitrogen [183]. It is evident that maintaining normal autophagy flux is crucial for sperm survival, and both autophagy deficiency and excessive autophagy can lead to sperm death. To attribute cell death to ADCD, the following criteria must be met: 1) there must be an elevation of autophagic flux during the cell death process; 2) the cell death process must be reversible through genetic or pharmacological inhibition of autophagy; 3) the death process must depend on at least two autophagy-related molecules, thereby excluding the possibility of individual molecules mediating cell death independently of autophagy; and 4) the death process must not be accompanied by other forms of cell death [182]. The elevation of autophagic flux and the rescue effect of inhibitors suggest that ADCD occurs in sperm freezing damage. However, CoQ_10_ can also rescue autophagy-mediated necrotic cells in human thawed sperm [142]. Therefore, the relationship between ADCD and sperm freezing damage requires further elucidation, particularly in distinguishing ADCD from AMCD during this process.

### Crosstalk between different types of RCD in sperm freezing damage

RCD is a major therapeutic target for various human diseases. However, the therapeutic results of inhibiting the initiation of a single RCD signal are sometimes unsatisfactory, which may be related to the highly interconnected nature of signaling modules of different types of RCD in addition to factors such as drug delivery and dose effects [3, 185–188]. Therefore, in the study of sperm freezing damage, it is not only necessary to distinguish which RCD is the primary one but also to clarify the crosstalk between different RCDs to maximize the quality of thawed sperm. Lipid peroxidation not only acts as an executor of ferroptosis but also induces apoptosis through its downstream products. The overloading of copper ions can induce both cuproptosis and ferroptosis. Necroptosis and apoptosis share a common upstream activation pathway, with Caspase 8 serving as a switch between pathways. Necroptosis can increase the intracellular ROS level, which can lead to lipid peroxidation and increase the risk of triggering ferroptosis. In addition, the connection between different types of RCD includes Ca^2+^ levels and autophagy.

#### Relationship between Ca^2+^ and various types of RCD

Intracellular Ca^2+^ homeostasis plays a crucial role in sperm, ensuring their normal physiological state and fertilization capability by regulating physiological functions closely related to sperm quality and male fertility potential, such as motility, fertilization, and the entire reproductive process. Besides massive cell death, the presence of a certain proportion of sperm in hyperactivated or acrosome-reacted states within thawed sperm is also a hallmark of sperm freezing damage. Although these states are necessary for sperm during the entire fertilization process, their premature occurrence can deplete energy and acrosomal enzymes, rendering sperm unable to effectively perform their tasks during fertilization and leading to fertilization failure. Such alterations in physiological state depend on the sperm’s ability to uptake extracellular Ca^2+^. Therefore, both depleting Ca^2+^ from the culture medium and adding the Ca^2+^ chelator EGTA to the thawing solution can enhance the fertilization capacity of thawed sperm [189, 190]. Within the female reproductive tract, the intracellular Ca^2+^ flux in sperm is tightly regulated by CatSper. An increase in intracellular Ca^2+^ flux, mediated by CatSper, can induce sperm hyperactivation, acrosome reaction, oocyte chemotaxis, and zona pellucida penetration during the fertilization process [191]. However, the expression of CatSper is reduced during sperm cryopreservation [192, 193], suggesting that the elevation of Ca^2+^ flux in cryopreserved sperm is independent of CatSper. Intriguingly, this may be associated with the development of some types of RCD.

Elevated Ca^2+^ influx resulting in morphological changes such as osmotic stress and cell rupture is a hallmark event in regulated necrosis, including necroptosis and ferroptosis [3]. MLKL forms a homotrimer through its amino-terminal coiled-coil domain, localizes to the cytoplasmic membrane during TNF-induced necroptosis, and activates downstream TRPM7 to mediate Ca^2+^ influx [194]. Similar to necroptosis, the increase in Ca^2+^ flux in cells undergoing ferroptosis precedes cell rupture, which is associated with the formation of nanoscale pores on the plasma membrane; however, the molecular mechanism underlying the formation of these pores remains unclear [147, 195, 196]. Recent studies show that treatment of sperm with different concentrations of the SLC7A11 inhibitor sulfasalazine (SS) significantly affects their motility [137]; in fresh horse sperm, low concentrations of SS enhance motility, exhibiting a phenomenon similar to hyperactivation, whereas high concentrations lead to decreased motility. However, for cryopreserved sperm, motility is reduced regardless of inhibitor concentration. Therefore, some phenotypic changes in cryopreserved sperm resemble the development of regulated necrosis, progressing from normal sperm to viable sperm with elevated intracellular Ca^2+^ flux without rupturing (undergoing hyperactivation, acrosome reaction, etc.) to dead sperm. Interestingly, intracellular Ca^2+^ overload can trigger endogenous apoptosis, potentially related to the mitochondria. Pretreatment of sperm with Ru360 to block Ca^2+^ entry into mitochondria reduces Caspase 3 activation and phosphatidylserine externalization induced by H_2_O_2_ stimulation [197]. Similarly, FAS-driven exogenous apoptosis is also associated with an increase in cytosolic Ca^2+^ [198–200].

#### Relationship between AMCD and various types of RCD

Autophagy determines the cellular fate of sperm under various environmental stresses. Exploring how autophagy induces other types of RCD and serves as a switch between different RCDs is crucial for understanding sperm freezing damage. The relationship between autophagy and apoptosis in sperm cryopreservation remains unclear. One possibility is that autophagy suppresses apoptosis, as activation of autophagy can enhance sperm motility, reduce the expression of mitochondrial outer membrane translocase TOMM20 and PINK1, and inhibit the activation of Caspase 3 and 7, thus reducing apoptosis and promoting cell survival [201]. Another possibility is that autophagy promotes apoptosis, which is a crucial step in the process of cell death, occurring mainly through two orderly mechanisms. First, autophagy directly induces cell death by phagocytosing apoptotic molecules or organelles such as mitochondria. In this process, specific autophagy-related proteins, like Fas-associated phosphatase 1 (Fap-1) and ATG5, interact with molecules in the apoptotic signaling pathway to regulate the initiation and execution of apoptosis. For instance, the degradation of Fap-1 enhances the activity of the Fas receptor, thereby promoting the transmission of apoptotic signals [202]; additionally, truncated fragments of ATG5 can directly act on mitochondria, driving the apoptotic process by disrupting mitochondrial function [203]. Second, autophagy molecules like ATG12 interfere with cellular survival mechanisms by directly binding to apoptotic molecules, thus triggering apoptosis [204]. This direct interaction impairs the function of anti-apoptotic proteins such as Bcl-2 and Mcl-1, relieving their inhibitory effect on apoptosis. This mechanism further intensifies the apoptotic tendency of cells, ensuring the smooth execution of the apoptotic program.

In necroptosis, the assembly and activation of the necrosome are crucial, with its core consisting of RIPK1, RIPK3, and MLKL. Autophagy machinery not only serves as a scaffold for the necrosome but also indirectly promotes the progression of necroptosis by degrading the apoptotic inhibitors c-IAP1 and c-IAP2 [205]. As sperm require a significant amount of energy to maintain their motility, sperm viability is generally positively correlated with ATP levels. Interestingly, autophagy can function as a switch between apoptosis and necroptosis, similar to Caspase 8, and this depends on the intracellular ATP content. When ATP reserves decrease, autophagy tends to trigger necroptosis, whereas when ATP is sufficient, autophagy may promote apoptosis [206, 207]. Additionally, autophagosome membranes and their associated proteins, such as p62, are also involved in this switching mechanism, regulating the localization and activity of molecules related to apoptosis and necroptosis and thereby facilitating the transition between different cell death modes [208].

Mitophagy may serve as one of the primary pathways for AMCD during sperm cryopreservation. Mitochondria, the primary source of energy for sperm, play a crucial role in maintaining sperm function through their quality control. Mitophagy, as an important means of quality control, is closely associated with sperm oxidative damage [209]. On one hand, mitophagy helps maintain mitochondrial homeostasis in sperm during cryopreservation, thereby suppressing apoptosis [201]. On the other hand, mitophagy may disrupt mitochondrial energy production, leading to the generation of excessive ROS in sperm during H_2_O_2_ incubation and the freeze-thaw process, which can then induce apoptosis and necrosis—the latter which can be rescued by CoQ_10_ [142]. Although the role of mitochondria in ferroptosis remains controversial, mitophagy may play a pivotal role in ferroptosis induction [210]. For instance, mitochondrial autophagy effectors such as PINK1 and DRP1 positively regulate ferroptosis [211]. However, the specific mechanisms underlying how mitochondrial autophagy affects the duration and intensity of lipid peroxidation in ferroptosis require further investigation. Additionally, mitochondrial fusion mediated by the fusion proteins MFN1 and MFN2 can also promote kinase-induced ferroptosis in certain situations [212], further highlighting the complexity of the interaction between mitochondrial autophagy and ferroptosis. In many cases, the occurrence of ferroptosis is highly dependent on autophagy mechanisms, including ferritinophagy, mitochondrial autophagy, and lipophagy. This type of ferroptosis is referred to as autophagy-dependent ferroptosis [213].

## Concluding remarks

The essence of sperm freezing damage is sperm death. This review summarizes the types of cell death that may occur after sperm freezing damage and analyzes the correlation between sperm freezing damage and ACD and RCD based on their characteristics. Overall, understanding the role of each type of RCD in sperm freezing damage may be the key to improving the quality of thawed semen. This review provides a feasible direction for future research on frozen semen.
